# Objective Methods to Assess Aorto-Iliac Calcifications: A Systematic Review

**DOI:** 10.3390/diagnostics14101053

**Published:** 2024-05-19

**Authors:** Anna Fornasari, Salomé Kuntz, Chiara Martini, Paolo Perini, Elisa Cabrini, Antonio Freyrie, Anne Lejay, Nabil Chakfé

**Affiliations:** 1 Vascular Surgery, Cardio-Thoracic and Vascular Department, Parma University Hospital, 43126 Parma, Italy; anna.fornasari91@gmail.com (A.F.); paolo.perini@unipr.it (P.P.); antonio.freyrie@unipr.it (A.F.); 2 Vascular Surgery, Kidney Transplantation and Innovation, Department of Vascular Surgery, University Hospital of Strasbourg, 67085 Strasbourg, Franceanne.lejay@chru-strasbourg.fr (A.L.);; 3 Gepromed, Medical Device Hub for Patient Safety, 67085 Strasbourg, France; 4 Department of Diagnostic, Parma University Hospital, 43126 Parma, Italy; 5 Department of Medicine and Surgery, University of Parma, 43126 Parma, Italy

**Keywords:** vascular calcification, aorto-iliac calcification, calcium score

## Abstract

Vascular calcifications in aorto-iliac arteries are emerging as crucial risk factors for cardiovascular diseases (CVDs) with profound clinical implications. This systematic review, following PRISMA guidelines, investigated methodologies for measuring these calcifications and explored their correlation with CVDs and clinical outcomes. Out of 698 publications, 11 studies met the inclusion criteria. In total, 7 studies utilized manual methods, while 4 studies utilized automated technologies, including artificial intelligence and deep learning for image analyses. Age, systolic blood pressure, serum calcium, and lipoprotein(a) levels were found to be independent risk factors for aortic calcification. Mortality from CVDs was correlated with abdominal aorta calcification. Patients requiring reintervention after endovascular recanalization exhibited a significantly higher volume of calcification in their iliac arteries. Conclusions: This review reveals a diverse landscape of measurement methods for aorto-iliac calcifications; however, they lack a standardized reproducibility assessment. Automatic methods employing artificial intelligence appear to offer broader applicability and are less time-consuming. Assessment of calcium scoring could be routinely employed during preoperative workups for risk stratification and detailed surgical planning. Additionally, its correlation with clinical outcomes could be useful in predicting the risk of reinterventions and amputations.

## 1. Introduction

Cardiovascular diseases (CVDs) remain a leading cause of morbidity and mortality worldwide, necessitating a thorough understanding of contributing factors for effective prevention and management [1]. Vascular calcifications in aorto-iliac arteries can significantly impact arterial compliance, hemodynamics, and overall cardiovascular health [2,3,4]. Accurate measurement of vascular calcifications in the aorta and peripheral arteries is imperative for comprehensive risk assessment, early diagnosis, and tailored intervention strategies [5]. While studies have explored the association between vascular calcifications and cardiovascular outcomes [6], there is no systematic review that consolidates the diverse methodologies utilized for their measurement. The existing literature offers fragmented insights into specific measurement techniques, potentially hindering a unified understanding of best practices in this domain. In addition, the existing literature is mostly based on the application of methodologies already in place for the measurement of coronary artery calcifications [7]. However, these methodologies do not always correlate with the same risk factors associated with calcifications found in the aorto-iliac region [8]. This systematic review aims to fill the existing gap in the literature by evaluating and summarizing the methods employed for measuring vascular calcifications in the aorto-iliac arteries and their correlation with CVDs and other eventual clinical outcomes.

## 2. Materials and Methods

A systematic literature review was conducted following the PRISMA Guidelines [9] (PRISMA Checklist available in the Supplementary Materials). The search encompassed all published items until 1 January 2024 on PubMed, Cochrane, and Medline databases, utilizing a combined search strategy involving the keywords “calcification AND aorta AND iliac”. Two investigators (A.F. and S.K.) screened all titles and abstracts retrieved from the search for relevance. Both reviewers independently obtained and reviewed the full texts of all relevant articles for suitability. Additionally, the reference lists of each article were reviewed for other potentially relevant studies. Any discrepancies in study inclusion were resolved through consensus.

### 2.1. Eligibility Criteria

All English studies reporting on methods to measure aorto-iliac calcifications were included. Non-English studies and those lacking exhaustive details on the calcification measurement method or uniformly applying the Agatston method for calcification index calculation have been excluded [7].

### 2.2. Data Items

Each reviewer independently collected data utilizing a standardized form. Any discrepancies in data extraction were resolved through consensus. The extracted data included study characteristics (author, year of publication, study design, and number of cases), along with a comprehensive description of the methodology employed and its correlation with clinical outcomes.

## 3. Results

**Figure 1:**
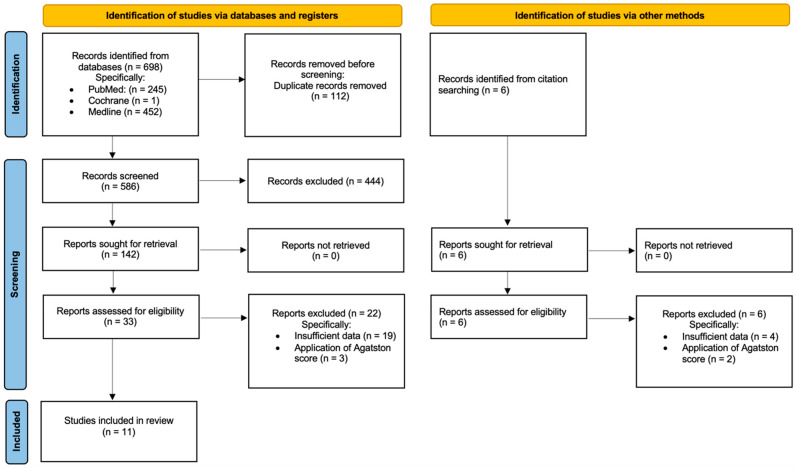
Flowchart showing study selection to review methods employed for measuring vascular calcifications in the aorto-iliac arteries.

The data extraction process involved the evaluation of 698 publications. After screening by title and abstract, 33 records were included for full-text examination. An additional six studies were identified through reference lists and related items. After full-text examination, 23 studies were excluded for insufficient data, and 5 articles were excluded because they applied calcification index calculation through an Agatston score [7]. No disagreements among the authors were reported during this process. Eleven publications were included. A descriptive presentation of the data was performed. Figure 1 displays the study selection flowchart. Table 1 exhibits the studies chosen for inclusion in the systematic review.

### 3.1. Calcification Measurement Methods

The results from the 11 selected studies (Table 1) revealed a diverse landscape of measurement methods for calcifications in the aorto-iliac arteries. Seven studies adopted manual measurement techniques [10,11,14,15,16,18,19], while the remaining studies explored automatic methods leveraging advanced technologies [12,13,17,20].

#### 3.1.1. Manual Measurement Methods

Ohya et al. [10] and Kimura et al. [19] described a manual method by analyzing abdominal plain computed tomography (CT): 10 slices of the abdominal aorta were obtained at 1 cm intervals from the aortic bifurcation. The area of the aortic cross-section and calcification was measured using image analysis software (ImageJ, version 1.45, National Institutes of Health, Bethesda, MD). The calcification area was measured and divided by the cross-sectional area to express the quantity of calcium as a percentage. The calcification index was calculated as the mean value of the percentage for the 10 slices. A similar method was applied in the study of Tsai et al. [11], where the calcified index was measured by the standard 64-multiple detector CT (MDCT) scan (LightSpeed VCT, GE Healthcare, Milwaukee, WI). The calcified area was calculated based on an attenuation range of >150 Hounsfield Units (HU) using image analysis software. The percentages of the area of the whole aorta affected by aortic calcification were calculated from the images of 4 consecutive slices just above the iliac bifurcation level. The study by Jayalath et al. [16] showed that the most appropriate threshold by which calcification should be measured was obtained by comparing clinical measurements taken on computed tomography angiography (CTA) and plain CT. Then, they defined the optimal threshold and performed the calcification volume measurements by using three different workstation protocols: (1) manual threshold setting without image magnification, (2) manual threshold setting with twice image magnification, and (3) automatic threshold setting with twice image magnification. Adrago et al. [18] described a simple vascular calcification score calculated on the pelvis and hand radiographic images. The X-rays were divided into sections and the presence of linear calcifications in each section was counted as 1 and its absence as 0. The final score was the sum of all the sections. NasrAllah et al. [15] compared calcium indices calculated through simple X-rays and CT scans of the thoracic, upper abdominal, and lower abdominal aorta. The calcium indices calculated using the X-rays either assigned scores for the presence of calcifications in the longitudinal aortic wall in lateral lumbar spine radiographs performed in standing position, or by applying the scoring system described by Adrago et al. [18]. Instead, the calcification index using plain CT abdominal scans was calculated by examining 10 consecutive cuts of the abdominal aorta at 1 cm intervals. Each cut was subdivided radially into 12 sectors. The number of sectors showing areas of calcification was counted and expressed as a percentage. Konjijn et al. [14] used 18F-FDG full-body positron emission tomography (PET)–CT for the evaluation of calcification encompassing all the vascular beds, including those from the abdominal aorta to plantar and dorsal arteries. The following imaging characteristics were scored. The severity of the calcifications was scored by a semi-quantitative scoring system divided into four categories: absent (no calcifications), mild (1–3 small calcifications), moderate (4–8 small, <3 large calcifications), and severe (>9 small, >3 large calcifications). Small calcifications are defined as calcifications that are visible on 1–2 slices, while large calcifications are visible on >3 slices. Annularity was scored as absent, dot(s), <90°, 90–270°, or 270–360°; thickness as absent, ≥1.5 mm or <1.5 mm; and continuity as indistinguishable, irregular/patchy or continuous. Moreover, the results from their imaging analysis were compared with results from a previous study by Kockelkoren et al. [21], who described a CT scoring method to distinguish intimal and medial internal carotid artery calcification with additional histological analysis. Annular, thin, and continuous calcifications correlated with media calcifications while dot-like, thick, and patchy calcifications correlated to intima calcifications.

#### 3.1.2. Automated or Semi-Automated Measurement Methods

Bagheri Rajeoni et al. [12] introduced a trained deep learning model designed to automatically analyze the vascular system from CTA scans. Following the extraction of the vascular system, calcifications are identified by the application of an intensity threshold to detect the number of pixels associated with calcifications, which are then measured in cubic volume through the application of a conversion factor. Their fully automated process calculates the lower extremity calcium scores within seconds. The performance of the deep learning model was assessed in terms of accuracy and reliability through performance analysis. They achieved an average Dice accuracy of 83.4% in segmenting arteries from the aorta to the knee, thereby improving upon the state of the art by 0.8% [22], with a correlation coefficient of 0.978. Additionally, they reported a Mean Absolute Percentage Error (MAPE) of 9.5% in measuring calcification compared to manual scoring.

Isgum et al. [13] applied an automatic method that extracts all connected objects above 220 HU from the CTA from the point where the superior mesenteric artery branches from the descending aorta until the first bifurcation of the iliac arteries. The calcifications were distinguished from non-calcifications by analyzing the object’s size, location, shape characteristics, and surrounding structures. Subsequently, the probability that an object represents a calcification is computed assuming a multivariate Gaussian distribution for the calcifications. Objects with low probability were discarded. The remaining objects are then classified into calcifications and non-calcifications using a 5-nearest neighbor classifier and sequential forward feature selection. Based on the total volume of calcifications determined by the system, the scan was assigned to one of those four categories: “no,” “small,” “moderate,” or “large” amounts of arterial calcification.

Kurugol et al. [17] created an automated pipeline for aorta segmentation that detects an initial aorta boundary by exploiting the cross-sectional circularity of the aorta in axial slices and aortic arch in reformatted oblique slices. This boundary was refined by a 3D level-set segmentation that evolved the boundary to the location of nearby edges. The next step was the detection of the aortic calcifications with thresholding and filtering out the false positive regions due to nearby high-intensity structures based on their anatomical location. Then, extraction of the centerline and oblique cross-sections of the segmented aortas were applied to compute the aorta morphology and calcification measures. These measures included volume, number of calcified plaques, and measures of vessel morphology such as average cross-sectional area, tortuosity, and width. Guidi et al. [20] measured calcification volumes from CTA extracted from the Picture Archiving and Communication Systems and Aquarius iNtuition Edition version 4.4.8 (TeraRecon Inc. San Mateo, California, USA). They previously developed an automatic method for the detection of the wall of the aorta and its main bifurcations based on an expert system method [23] combined with supervised deep learning [22]. The method consists of 6 sequential steps. After image pre-processing using a median filter, the spine and the vessel’s lumen are segmented using the expert system method. A trained convolutional neural network is then applied to refine the detection of the vascular wall. The intraluminal thrombus is segmented using an Active Contours Without Edges algorithm.

Vascular calcifications are then segmented by applying a threshold within a ring-shaped working region defined using a morphological erosion operator. The method analyzes each slice following the axial axis within a segmentation mask obtained from the contour of the artery wall. A morphological dilate operator is applied to the aorta mask and a morphological erode of the same size, which are then subtracted from the original mask. The working region is therefore reduced to a ring-shaped region to search for calcifications. 

Finally, a threshold is applied in the ring-shaped working region. All the volume elements, or voxels, with HU above the threshold, are defined as calcifications. As the quality of the CTAs can be heterogeneous depending on the manufacturer or the protocol used for contrast media administration, automatic estimation of the threshold has been added within the pipeline to adjust the frequency of the HU intensity in the aorta pixels to identify calcification. The maximal frequency is used as a basis for the relative selection of the threshold. The upper HU intensity corresponding to a frequency of 5% of the maximal frequency is used as the threshold to extract the calcifications. Calcification volumes were measured in the infrarenal abdominal aorta and the common and external iliac arteries. This method allowed an automatic quantification of vascular calcifications in a selected region including number, individual, and total volumes.

### 3.2. Clinical Outcomes

Within the 11 studies included in the review, 6 studies included clinical outcomes correlated to the calcium score [10,11,15,18,19,20]. Ohya et al. [10] divided the patients into two groups according to their calcium score being lower or higher than the mean value. Among the 137 patients enrolled, the calcium index ranged from 0% to 57.6%, with a mean of 20.7 ± 15.3%. The risk factors in each group were compared by multivariate analysis and logistic regression to identify independent risk factors for aortic calcification. Age (high calcium group: 64.7 ± 9.9 years vs. low calcium group 54.4 ± 11.6 years, *p* < 0.001), systolic blood pressure (high calcium group: 148.6 ± 14.7 mmHg vs. low calcium group: 138.2 ± 14.7 mmHg, *p* < 0.001), serum calcium (high calcium group: 9.63 ± 0.43 mg/dL vs. low calcium group: 9.34 ± 0.42 mg/dL, *p* < 0.047), and lipoprotein(a) levels (high calcium group: 29 ± 19.5 mg/dL vs. low calcium group: 19 ± 17 mg/dL) were independent risk factors for aortic calcification. Tsai et al. [11] investigated the CV mortality and non-CV mortality during the follow-up period (6.8 years, IQR = 3.6–9.2) using the Cox proportional hazard model and time-dependent receiver operating characteristic (ROC) analysis. The abdominal aorta calcification ratio (AAC) was significantly higher in the CV mortality group compared with others without CV mortality (32.6 ± 18.6% vs. 12.9 ± 15.3%, *p* < 0.001). In time-dependent ROC analysis, AAC had excellent predictive power of CV mortality (AUC: 0.787) but not non-CV mortality (AUC: 0.537).

The best cutoff value for the AAC ratio was 39% to predict CV mortality (hazard ratio, 8.01; 95%CI = 3.14–20.44). NasrAllah et al. [15] showed CT scans of the upper and lower abdominal aorta detected more calcification than plain lateral lumbar X-ray (*p* = 0.004, Chi-square = 8.4 vs. *p* < 0.001, Chi-square = 26.5). Moreover, the qualitative and quantitative presence of calcification increased from proximal to distal segments (lower abdominal aorta > upper abdominal aorta > thoracic aorta). The presence of calcification in the distal aorta and pelvic vessels had the highest predictive value for CV events and mortality. In the cohort of hemodialysis patients included in the studies by Adrago et al. [18] and Kimura et al. [19], diabetes, male sex, age, duration of hemodialysis, mean arterial pressure, coronary artery disease (CAD), and peripheral arterial disease (PAD) were independently associated with a higher calcium index. Guidi et al. [20] aimed to evaluate if vascular calcification volumes could predict the risk of target lesion reintervention (TLR) in patients with aorto-iliac occlusive disease (AIOD) who underwent endovascular revascularization. They retrospectively included 117 patients, for a mean follow-up of 39 months. TLR was performed in 55 patients (32.2%). No significant difference was observed between the group of patients who underwent TLR compared to patients who did not in terms of general characteristics of patients including age, sex, or cardiovascular comorbidities. However, patients who had a re-intervention had significantly higher volumes of calcification in the right and left iliac arteries (right iliac artery: 2.274 vs. 1.606 mm^3^, *p* = 0.0319; left iliac artery: 2.278 vs. 1.567 mm^3^, *p* = 0.0213).

## 4. Discussion

A growing interest is developing in understanding vascular calcification and its correlation with risk factors and subsequent outcomes [24,25]. Research focused on coronary arteries reveals a clear link between calcification and cardiovascular events, particularly myocardial infarction [26]. Studies examining coronary circulation indicated a direct correlation between the extent of calcification, the severity of atherosclerosis, and the occurrence of clinical events [27]. This relationship extends beyond coronary arteries, as similar associations between calcification and cardiovascular events have been observed in other vascular regions, including the aortic arch and the thoracic aorta [28].

However, compared to investigations of coronary circulation, very few studies have assessed the ability of abdominal aortic calcification to predict CV events [29]. In the field of cardiology, significant progress has been made in developing methods to measure calcifications. The Agatston score is commonly utilized as the most robust test for further atherosclerotic CVD risk stratification [7,30]. However, the Agatston score may not accurately reflect the severity or implications of calcifications in vessels outside the heart. There are important differences that should be accounted for, such as vessel size and wall thickness, hemodynamics, and the presence of different surrounding organs [31].

**Figure 2:**
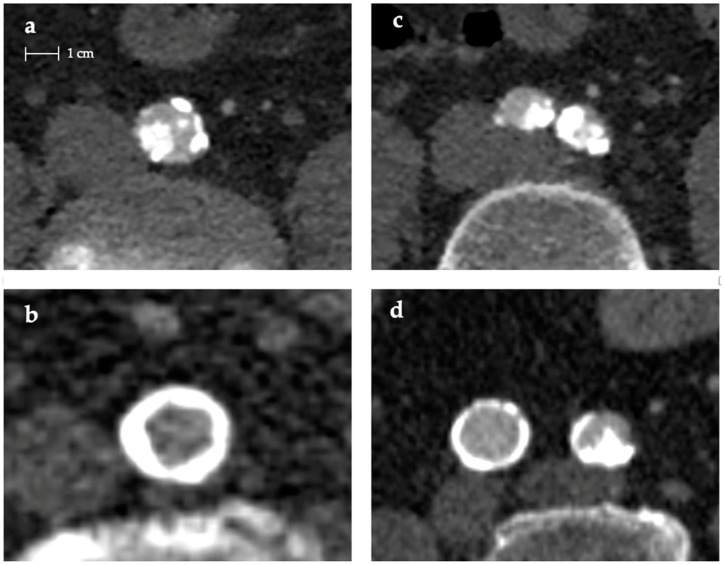
Different aorto-iliac plaques’ morphology: (**a**) irregular, protruding aortic plaque; (**b**) aortic circumferential plaque; (**c**) irregular and protruding bilateral iliac plaques; (**d**) circumferential plaque in right iliac, irregular, and protruding plaque in left iliac.

For a more comprehensive understanding of vascular calcification outside the coronary bed, this review focused on the current methods available to assess aorto-iliac and peripheral vessel calcification. The variation of imaging methods used to quantify calcification in these studies (plain x-ray, PET-CT, plain CT, and CTA) and the lack of rigorous assessment of reproducibility makes interpretation of the available data difficult. The value of using CTA to carry out such quantification rather than plain CT is that both angiography and calcification data can be obtained in one image run, thereby reducing the dose of ionizing radiation to the patient and providing cost savings [16]. However, as underlined by Buijs et al. [31], calcification scoring on CTA tends to overestimate volume and mass suggesting a low accuracy and reliability. These are reduced further by the interference of intravascular contrast. As described by Kurugol et al. [17], the advantage of measuring calcifications on CTA lies in its ability to offer supplementary information on vessel morphology, such as average cross-sectional area, tortuosity, and width. This is accomplished through the automatic computation of a center lumen line. Thus, it not only provides insights into how calcifications impact the vessel lumen but also understanding other aspects of its morphology. Figure 2 shows different plaque morphology.

These characteristics can significantly impact surgical planning by impairing the outcome of angioplasty or stenting, as the presence of calcium can impair the access or the treatment of the arterial lesions due to difficulties in inserting the wires or dilating the vessel [20,32,33,34]. Yin et al. [35] used optical coherence tomography (OCT) to predict newly implanted stent expansion for the treatment of in-stent restenosis (ISR) in coronary arteries. New stent underexpansion was found in lesions that had increased neointimal or peri-stent calcium compared to those without new stent underexpansion (69.7% vs. 37.3%, *p* = 0.001). Moreover, a maximum calcium angle >180° and a maximum calcium thickness >0.5 mm were independently associated with new stent underexpansion.

Most of the studies included in this review describe manual approaches for evaluating vascular calcifications, characterized by time-consuming, subjective, and error-prone processes. Automatic methods, leveraging artificial intelligence technologies, exhibit promising outcomes by delivering comparable information in a more efficient manner and accommodating larger datasets. As described by Fischer et al. [36], the basic foundations of artificial intelligence (AI) include the following: (1) analyzing large amounts of data, (2) recognizing patterns, (3) predicting outcomes, and (4) aiding in drawing conclusions to improve workflows. Vascular surgery especially depends substantially on diagnostic imaging and large amounts of patient data. The ability of AI to analyze those data, detect patterns, and draw conclusions surpasses human capacities and has already proven beneficial to patient treatment and outcomes. AI has brought new insights into cardiovascular imaging and several studies proposed the use of machine learning algorithms to develop automatic coronary artery calcium scoring [37,38,39]. The automated method described by Bagheri Rajeoni et al. [12], underscores the potential of deep learning techniques as a rapid and accurate tool to assess calcification in the abdominal aorta and its branches above the patella.

The study by Guidi et al. [20] correlates higher calcium scores with adverse events after revascularization, notably TLR. Other studies in the literature demonstrated these results, correlating higher calcium burden with earlier loss of patency and major adverse limb events (MALEs), which include reintervention and major limb amputation [40,41].

Calcifications have also been identified as a risk factor for mortality and morbidity in patients with PAD. In a Cox proportional hazard model with lower extremity artery calcifications divided into quartiles, patients with PAD with the highest quartile of calcification score had a 5.16-fold risk for all-cause mortality [42]. In addition, patients with complete annular calcifications had a higher all-cause 10-year mortality (hazard ratio adjusted for age and sex = 1.68, *p* = 0.04) [14]. Finally, a high lower limb arterial calcification score in patients with symptomatic PAD was significantly associated with ischemic heart disease (*p* = 0.028) and all-cause mortality (*p* = 0.012) [43]. In the study by Konijn et al. [14], a correlation between CT and histological characteristics of calcifications was found. Annular, thin, and continuous calcification correlated with media calcifications while dot-like, thick, and patchy calcifications correlated to intima calcifications. These two patterns of vascular calcification probably represent two different diseases. Intimal calcifications are related to atherosclerotic disease and are influenced by risk factors such as dyslipidemia, hypertension, and smoking [44]. Medial calcifications frequently are annular and lead to a stiff vessel wall and increasing pulse pressure. Medial calcifications are probably caused by a disbalance between pro- and anti-calcifying agents and are found in chronic kidney disease, diabetes, and aging [45,46]. This suggests that distinguishing between various calcifications is essential, as they may be associated with different risk factors and therefore need to be treated and prevented in different ways.

We believe a comprehensive calcification measurement method should include the following key characteristics: it should be automatically computed using AI to ensure efficiency and broad applicability for risk stratification, without being time-consuming; it should analyze CTA images to help in decision making and facilitate accurate surgical planning by providing information on plaque morphology and its impact on the vessel’s lumen. The method should also extend to follow-up imaging, predicting the risk of TLR and MALEs. This facilitates the establishment of personalized follow-up protocols, contributing to a more comprehensive and personalized patient care approach.

### Limitations

The main limitation of this study is the literature reviewed spans diverse methodologies and imaging techniques, leading to heterogeneity in the data. The variation in calcification assessment methods, including plain X-ray, PET-CT, plain CT, and CTA, introduces challenges in comparing the different results. Moreover, the lack of standardized reproducibility assessment across these studies raises concerns about the reliability of the reported data.

## 5. Conclusions

This review sheds light on the evolving landscape of vascular calcification assessment. The focus on arterial calcium burden from a quantitative point of view provides a foundation, but there is a pressing need for tools to identify additional plaque characteristics, such as shape and sharpness. The utilization of AI, owing to its broad applicability, holds promise in capturing additional qualitative characteristics of plaques such as shape, location, and distribution, thereby enriching our understanding of vascular calcifications. Identifying and standardizing measurement methods has the potential to provide risk stratification, influence surgical planning, and significantly contribute to predicting clinical outcomes, especially regarding reinterventions and MALEs.

The journey to a more comprehensive understanding of vascular calcification is ongoing, with emerging technologies and methodologies poised to play a pivotal role in advancing research and clinical applications in the field of vascular surgery.

## Figures and Tables

**Table 1:** Summary of studies chosen for systematic review on objective methods to assess aorto-iliac calcification.

Author Journal Year	Study Design	Number of Cases	Imaging Modality	Automated/Manual	ROI	Methodology	Outcomes
Ohya et al. [10] *Inter Med* 2010	Prospective observational	137	CT	Manual	Abdominal aorta	10 slices at 1 cm intervals from the aortic bifurcation. The calcification area is divided by the cross-sectional area and expressed as a percentage.	Risk factors for abdominal aortic calcification in HD patients include age, systolic blood pressure, and serum calcium.
Tsai et al. [11] *Medicine* 2020	Prospective observational	123	CT	Manual	Abdominal aorta	The percentages of the area of the whole aorta affected by aortic calcification were calculated from the images of 4 consecutive slices just above the iliac bifurcation level.	Aortic calcification ratio (volume of calcific aorta/total aortic volume) has excellent prognostic value of CV mortality but is unable to predict non-CV mortality.
Bagheri Rajeoni et al. [12] *Diagnostic* 2023	Prospective observational	27	CTA	Automated	From descending thoracic aorta to the patella	Trained deep learning model to automatically extract vascular system. Through intensity thresholding, the number of pixels associated with calcifications is measured. A conversion factor is applied to measure calcification volume.	The method achieved 83.4% average Dice accuracy in segmenting arteries from the aorta to the patella, advancing the state of the art by 0.8%.
Isgum et al. [13] *Acad Radiol* 2004	Prospective observational	40	CTA	Automated	From first slice below SMA ostium to the first slice above the iliac arteries bifurcation	Extraction of all objects above 220 HU from the scan. Objects with low probability of being calcifications are discarded. Objects are then classified into calcifications and non-calcifications using a 5-nearest neighbor classifier. Based on the total volume of calcifications, the scan is assigned to one of the four categories: “no,” “small,” “moderate,” or “large” amounts of arterial calcification.	It is possible to identify most arterial calcifications in abdominal CT scans in a completely automatic fashion with few false positive objects, even if the scans contain contrast material.
Konijn et al. [14] *Eur J Radiol* 2020	Retrospective	204	PET-CT	Manual	Abdominal aorta and lower limbs	Scoring characteristics: (1) severity: absent (no calcification), mild (1–3 small calcification), moderate (4–8 small or < 3 large calcifications), severe (>9 small or >3 large calcifications); (2) annularity: absent, dot(s), <90°, 90–270° or 270–360°; (3) thickness: absent, ≥1.5 mm or < 1.5 mm; (4) continuity: indistinguishable, irregular/patchy, or continuous.	Correlation between annular, thin, and continuous calcification characteristics and media calcifications while dot-like, thick, and patchy calcifications correlate to intima calcifications.
NasrAllah et al. [15] *Int J Cardiol* 2016	Prospective observational	111	CT vs. X-Ray	Manual	Thoracic aorta, abdominal aorta, iliac arteries	Application of 6 vascular calcification scores: 2 scores utilized simple X-rays of abdominal aorta and peripheral vessels; 4 scores used CT scans of the thorax, abdomen, and pelvis to calculate the calcification index.	CT-based techniques are more sensitive than plain X-rays at detecting peripheral and aortic vascular calcifications.
Jayalath et al. [16] *Arterioscler Thromb Vasc Biol* 2006	Prospective observational	50	CT vs. CTA	Manual	Abdominal aorta	CT and CTA analyzed using 5 different thresholds to define aortic calcification. CTA analysis for calcification volume measurements using three different protocols: (1) manual threshold setting without image magnification, (2) manual threshold setting with twice image magnification, and (3) automatic threshold setting with twice image magnification.	CTA provides accurate data on calcifications and in predicting subsequent events which, in addition to coronary events, may also include aorticocclusion, aneurysm formation, and outcome of interventional procedures on the aorta.
Kurugol et al. [17] *Med Phys* 2015	Prospective observational	2500	CTA	Automated	Aortic arch	Algorithm: (1) detection of aorta boundary, (2) detection of aortic calcifications with thresholding, (3) extraction of the centerline of the segmented aortas to compute the aorta morphology and calcification measures. Measures include volume and number of calcified plaques and measures of vessel morphology such as average cross-sectional area, tortuosity, and arch width.	Development of an objective tool to assess aorta morphology and aortic calcium plaques on CT scans that may be used to provide information about the presence of cardiovascular disease and its clinical impact.
Adragao et al. [18] *Nephrol Dial Transplant* 2004	Prospective observational	123	X-Ray	Manual	Pelvis and hand	Pelvis X-rays were divided into four sections while hand X-rays were divided into two sections. The presence of linear calcifications in each section was counted as 1 and its absence as 0. The final score was the sum of all the sections, ranging from 0 to 8.	Extensive vascular calcifications may represent one of the factors contributing to the extremely high CV mortality for HD patients when compared with the general population.
Kimura et al. [19] *Kidney Int Suppl* 1999	Prospective observational	132	CTA	Manual	Abdominal aorta	10 slices of the abdominal aorta at 1 cm intervals from the aortic bifurcation. For each slice, calculation of area of calcification over cross-sectional area. Total calcification area divided by total cross-sectional area to calculate calcification extent as a percentage.	Correlation between higher systolic blood pressure and serum Ca and Pi with severity of abdominal aorta calcification.
Guidi et al. [20] *Ann Vasc Surg* 2022	Retrospective	171	CTA	Automated	Infrarenal abdominal aorta, left and right common, and the external iliac arteries	Three sequential steps: (1) image pre-processing, (2) lumen segmentation using expert system, (3) deep learning algorithms and segmentation of calcifications. Automatic quantification of vascular calcifications in a selected region including number, individual, and total volumes.	Target lesion re-intervention was performed in 55 (32.2%) patients who had higher volume of calcifications in the iliac arteries, compared with patients who did not have a reintervention. The development of fully automatic software would be useful to facilitate the measurement of vascular calcifications and possibly better inform the prognosis of patients.

ROI: region of interest, CT: plain computed tomography, HD: hemodialysis, CV: cardiovascular, CTA: computed tomography angiography, HU: Hounsfield units, PET-CT: positron emission tomography–computed tomography, Ca: serum calcium, Pi: serum phosphate.

## Data Availability

Not applicable.

## Supplementary Materials

The following supporting information can be downloaded at: https://www.mdpi.com/article/10.3390/diagnostics14101053/s1, PRISMA Checklist.
